# Validations of Top and Novel Susceptibility Variants in All-Age Chinese Patients With Acute Lymphoblastic Leukemia

**DOI:** 10.3389/fgene.2020.01004

**Published:** 2020-08-25

**Authors:** Fei Liao, Yuanxin Ye, Dandan Yin, Yun Qin, Jiangyan Zhao, Wanhua Zhang, Yan Zhang, Zhujun Deng, Yuelan Wang, Binwu Ying, Lanlan Wang, Ju Gao, Yang Shu, Yiping Zhu, Xiaoxi Lu

**Affiliations:** ^1^Key Laboratory of Birth Defects and Related Diseases of Women and Children, Department of Pediatric Hematology/Oncology, West China Second Hospital, Sichuan University, Ministry of Education, Chengdu, China; ^2^State Key Laboratory of Biotherapy, Department of Laboratory Medicine, Precision Medicine Center, West China Hospital, Sichuan University, Chengdu, China; ^3^Department of Radiology, West China Hospital, Sichuan University, Chengdu, China; ^4^Department of Hematology and Hematology Research Laboratory, West China Hospital, Sichuan University, Chengdu, China; ^5^State Key Laboratory of Biotherapy, Department of Thoracic Oncology, Cancer Center, West China Hospital, Sichuan University, Chengdu, China

**Keywords:** acute lymphoblastic leukemia, genetic susceptibility, *ERG*, all-age patients, subtype specific

## Abstract

Through genome-wide association studies (GWAS), multiple inherited predispositions to acute lymphoblastic leukemia (ALL) have been identified in children. Most recently, a novel susceptibility locus at *ERG* was localized, exhibiting Hispanic-specific manner. In this study, we conducted a replication study to in all-age Chinese patients (*N* = 451), not only validating the novel *ERG* locus, but also systematically determining the impact of age on association status of the top GWAS signals. We found that single nucleotide polymorphisms at *ARID5B*, *IKZF1*, *CEBPE*, *PIP4K2A* were only significantly associated with ALL susceptibility in childhood patients with no *BCR-ABL* fusion, while *GATA3* signal exhibited its significance in adults no matter carrying *BCR-ABL* fusion or not. Moreover, the novel *ERG* SNP can be validated in pediatric patients without both *BCR-ABL* and *ETV6-RUNX1* fusion. Our finding suggests the modifying effects of age on genetic predisposition to ALL, and highlights the impact of *ERG* SNP in Chinese patients.

## Introduction

Acute lymphoblastic leukemia (ALL) is a leading cause of disease-induced death, especially in children aged between 2 and 5 years old, and age is negatively related to prognosis and survival rates of ALL patients according to the clinical statistics. The inferior prognosis of adult ALL patients is likely to be multifactorial, including the age-related differences in leukemia blasts and host genomic alterations. Leukemogenesis mechanisms were considered to be different between children and adults. For example, more frequent somatic *BCR-ABL* fusion (also known as Philadelphia chromosome positive, Ph^+^) were observed in adults’ (Inaba et al., 2013). Additionally, racial disparities were also noticed in terms of incidence and treatment outcomes, highlighting the importance of age- and ethnicity- related study on leukemogenesis and clinical practice.

By conducting genome-wide association studies (GWASs), we and other independent groups identified multiple top inherited predispositions to acute lymphoblastic leukemia (ALL) susceptibility, including single nucleotide polymorphisms (SNPs) at *ARID5B*, *IKZF1*, *GATA3* and etc. (Papaemmanuil et al., 2009; Trevino et al., 2009; Perez-Andreu et al., 2013, 2015; Xu et al., 2013, 2015; Vijayakrishnan and Studd, 2018; Wiemels et al., 2018). Association of these SNPs are greatly impacted by clinical characteristics (e.g., age, ethnicity, and subtypes) (Xu et al., 2012; Perez-Andreu et al., 2015; Liao et al., 2016). For instance, we and another independent group noticed that association of *ARID5B* and *GATA3* SNPs with ALL risk were oppositely influenced by age in Caucasians and Hispanics (Migliorini et al., 2013; Xu et al., 2013). Also, we recently identified a novel locus at rs2836365 in *ERG* gene, which tends to exhibit Hispanic specific manner and varied in different subtypes (Qian et al., 2018). This novel site has been recently validated in independent studies (de Smith et al., 2019; Vijayakrishnan et al., 2019), but predominately performed in childhood without BCR-ABL fusion, with limited systematical investigation in Ph^+^ adolescents/adults and Chinese patients.

In this study, we sought to investigate association of the reported top GWAS signals (i.e., *ARID5B*, *IKZF1*, *GATA3*, *PIP4K2A*, *CEBPE*, and *ERG*) with ALL susceptibility in all-age Chinese patients, and also estimate the impact of BCR-ABL fusion and age at diagnosis.

## Materials and Methods

### Subjects and Genotyping

Peripheral blood was obtained from 456 non-ALL individuals, as well as 451 all-age B-lineage ALL patients, who were treated with standard protocol in Department of Hematology/Oncology, West China Hospital and West China Second Hospital (e.g., CCGC-ALL2015, registered in http://www.chictr.org.cn/with ID: ChiCTR-IPR-14005706) (Zhu et al., 2018; Cao et al., 2020). Clinical information of each patient was obtained from the electronic records system at our hospitals, including gender, age and white blood cell (WBC) at diagnosis, molecular subtypes. Fusion-based molecular subtypes were determined by Fluorescence *in situ* hybridization, while hyperdiploid was determined by flow cytometry-based DNA index. Patients (14 years is considered as children, while the rest were considered as adolescents/adults (following referred as “Adults”) in this study.

Nine SNPs at 6 loci (ARID5B, IKZF1, GATA3, PIP4K2A, CEBPE, and ERG) were directly genotyped through Sanger sequencing. Additionally, we also retrieved genotype information of a large Chinese Han population from the public dataset to increase the statistical power (Cai et al., 2017). After filtered out the individuals with missing information of either SNPs analyzed in this study, genotype information of 10,640 out of 11,670 individuals from this public database were used for further association analyses. Comparison were conducted between these two control cohorts in terms of risk allele frequencies (RAF). Because the prevalence of patients with ALL is less than 1 in 10,000 in China, these two control cohorts were combined and considered as non-ALL controls (totally 11,096) for SNPs with no significant difference.

This study was approved by Ethnics Committee of West China Hospital and West China Second Hospital, and informed consent was obtained from patients or their guardians, as appropriate.

### Statistical Analysis

All SNPs have passed the quality control based on call rate of the SNPs (at least 95% patients have been successfully genotyped for each SNP), and Hardy-Weinberg equilibrium (*P* > 0.05 for χ^2^-test). The association of each SNP with ALL susceptibility was estimated by comparing the genotype frequency between ALL cases and non-ALL controls by using logistic regression model. *P*-value, odds ratio (OR) and 95% confidence interval (95% CI) was estimated by using R (version 3.5), and a two-sided *P* < 0.05 was considered as statistically significant.

## Results

Totally 451 B lineage all-age ALL patients were included in this study, and were subsequently divided into two age groups, including 294 childhood (median age = 4.5; ≤ 14 years), and 157 adolescents/adults (median age = 37; range 14–68). Baseline characteristics of ALL patients are summarized in Table 1. BCR-ABL fusion has been taken it into account in the association test, because it is enriched in adults and barely considered in previous association studies. Totally nine SNPs were directly genotyped in all cases and 456 non-ALL controls, with overall call rate >95%. To increase the statistical power, genotypes of these nine SNPs were also retrieved from public dataset with 10,640 Han Chinese, and compared with our non-ALL controls in terms of risk allele frequencies (RAF). No significantly difference were observed in any of these SNPs, we therefore combined the two control cohorts (*N* = 11,096) to conduct association analyses (Table 2).

**TABLE 1:** Characteristics of patients.

Clinical features	Children (*N* = 294)	Adults (*N* = 157)
Age at diagnosis (years); median ± *SD*	4.5 ± 2.98	37 ± 13.35
**Gender**		
Male, *N* (%)	152 (51.7%)	90 (57.3%)
Female, *N* (%)	142 (48.3%)	67 (42.7%)
WBC (× 10^9^/L); median ± *SD*	10.65 ± 67.79	17.03 ± 96.73
**Molecular subtype**		
*ETV6-RUNX1*	64	1
*TCF3-PBX1*	12	2
*BCR-ABL* (Ph^+^)	16	91
Hyperdiploid*	32	0

*Hyperdiploid subtype was not determined in 149 out of 451 patients, because it is not routinely tested before 2016, especially in adults.

**TABLE 2:** Comparison of two control cohorts.

SNP ID	Gene	RAF of controls		*P*-value
		In-House (*N* = 456)	Public control (*N* = 10,640)	
rs10821936	*ARID5B*	0.366	0.357	0.65
rs7090445	*ARID5B*	0.364	0.359	0.86
rs3824662	*GATA3*	0.306	0.318	0.56
rs11978267	*IKZF1*	0.1	0.146	0.23
rs11770117	*IKZF1*	0.853	0.846	0.73
rs4982731	*CEBPE*	0.175	0.147	0.13
rs4748793	*PIP4K2A*	0.815	0.809	0.6
rs7088318	*PIP4K2A*	0.614	0.589	0.91
rs2836365	*ERG*	0.262	0.272	0.29

*RAF, risk allele frequency.*

After comparing the genotype frequency between ALL cases and non-ALL controls, six SNPs at 4 loci (i.e., *GATA3*, *ARID5B*, *IKZF1*, and *CEBPE*) were significantly associate with ALL susceptibility in all-age patients (Table 3). rs10821936 at *ARID5B* locus [*P* = 1.8 × 10^–11^, OR = 1.59 (1.39–1.81)] was in high lineage disequilibrium (*r*^2^ = 0.98) with rs7090445 [*P* = 5.3 × 10^–10^, OR = 1.53 (1.34–1.76)], which was considered to be the potential causal variant for *ARID5B* locus (Studd et al., 2017), whereas rs11770117 [*P* = 0.003, OR = 1.41 (1.12–1.76)] at *IKZF1* exhibit as an independent signal even after adjust for rs11978267 [*P* = 0.005, OR = 1.32 (1.09–1.59)] with *P*_adjust_ = 0.02, which is consistent with our previous observation in Hispanics (Xu et al., 2013). when BCR-ABL fusion was taken into accounts, SNPs at *ARID5B*, *IKZF1* and *CEBPE* loci lost their significance in Ph^+^ patients, while risk allele of rs3824662 at *GATA3* locus was overrepresented in both Ph^+^ patients [*P* = 0.02, OR = 1.40 (1.07–1.83)] and Ph^–^ patients [*P* = 0.04, OR = 1.18 (1.01–1.38)] (Table 3).

**TABLE 3:** Association of the GWAS hits with ALL susceptibility in all-age Chinese patients.

SNP ID	Genes	Location (Chr:Position)	Allele	Subtype group	All age			Childhood (≤14 years)			Adults (>14 years)			Control
					RAF	*P*-value	OR (95% CI)	RAF	*P*-value	OR (95% CI)	RAF	*P*-value	OR (95% CI)	RAF
rs10821936	*ARID5B*	10:63723577	C*/T	all	0.47	**1.8 × 10^–11^**	1.59 (1.39–1.81)	0.51	**1.8 × 10^–13^**	1.86 (1.58–2.19)	0.39	0.17	1.17 (0.93–1.47)	0.36
				Ph^–^	0.49	**2.7 × 10^–12^**	1.73 (1.49–2.02)	0.51	**1.7 × 10^–13^**	1.91 (1.61–2.26)	0.39	0.39	1.17 (0.82–1.66)	
				Ph^+^	0.39	0.36	1.14 (0.86–1.50)	0.34	0.87	1.06 (0.51–2.20)	0.4	0.29	1.18 (0.87–1.58)	
rs7090445	*ARID5B*	10:63721176	C*/T	all	0.46	**5.3 × 10^–10^**	1.53 (1.34–1.76)	0.51	**2.5 × 10^–13^**	1.91 (1.60–2.26)	0.37	0.59	1.07 (0.85–1.34)	0.36
				Ph^–^	0.49	**1.4 × 10^–11^**	1.71 (1.46–1.99)	0.52	**1.7 × 10^–13^**	1.91 (1.61–2.26)	0.38	0.68	1.08 (0.76–1.54)	
				Ph^+^	0.37	0.8	1.04 (0.78–1.37)	0.34	0.85	1.07 (0.52–2.22)	0.37	0.72	1.06 (0.78–1.43)	
rs3824662	*GATA3*	10:8104208	A*/C	all	0.37	**0.002**	1.24 (1.09–1.43)	0.35	0.1	1.15 (0.97–1.36)	0.4	**0.001**	1.44 (1.15–1.80)	0.32
				Ph^–^	0.36	**0.04**	1.18 (1.01–1.38)	0.34	0.21	1.12 (0.94–1.33)	0.41	**0.03**	1.47 (1.04–2.06)	
				Ph^+^	0.4	**0.02**	1.40 (1.07–1.83)	0.38	0.5	1.28 (0.63–2.58)	0.4	**0.02**	1.42 (1.06–1.90)	
rs11978267	*IKZF1*	7:50398606	C*/T	all	0.17	**0.005**	1.32 (1.09–1.59)	0.18	**0.002**	1.39 (1.12–1.72)	0.15	0.68	1.09 (0.73–1.63)	0.14
				Ph^–^	0.18	**0.004**	1.36 (1.10–1.68)	0.18	**0.001**	1.44 (1.16–1.79)	0.12	0.66	1.17 (0.58–2.34)	
				Ph^+^	0.15	0.6	1.13 (0.72–1.78)	0.1	0.57	1.41 (0.43–4.64)	0.16	0.38	1.25 (0.76–2.04)	
rs11770117	*IKZF1*	7:50406065	A*/T	all	0.89	**0.003**	1.41 (1.12–1.76)	0.88	**0.02**	1.36 (1.06–1.76)	0.9	0.06	1.55 (0.98–2.47)	0.85
				Ph^–^	0.89	**0.006**	1.42 (1.11–1.83)	0.88	**0.02**	1.39 (1.06–1.81)	0.91	0.17	1.73 (0.79–3.76)	
				Ph^+^	0.88	0.29	1.31 (0.80–2.14)	0.83	0.84	1.10 (0.42–2.87)	0.89	0.2	1.46 (0.82–2.59)	
rs4982731	*CEBPE*	14:23585333	C*/T	all	0.19	**0.008**	1.35 (1.08–1.68)	0.2	**0.007**	1.40 (1.09–1.79)	0.17	0.52	1.17 (0.72–1.92)	0.15
				Ph^–^	0.19	**0.02**	1.36 (1.06–1.74)	0.2	**0.009**	1.41 (1.09–1.82)	0.13	0.72	1.21 (0.43–3.43)	
				Ph^+^	0.15	0.87	1.05 (0.62–1.78)	0.04	0.18	3.94 (0.53–28.9)	0.19	0.32	1.32 (0.76–2.31)	
rs4748793	*PIP4K2A*	10:22483011	A*/G	all	0.84	0.13	1.20 (0.95–1.51)	0.84	0.1	1.25 (0.96–1.63)	0.82	0.86	1.04 (0.65–1.66)	0.81
				Ph^–^	0.84	0.12	1.23 (0.95–1.60)	0.85	0.07	1.30 (0.98–1.72)	0.76	0.52	1.29 (0.60–2.79)	
				Ph^+^	0.82	0.84	1.05 (0.65–1.71)	0.75	0.47	1.39 (0.57–3.41)	0.84	0.53	1.20 (0.67–2.16)	
rs7088318	*PIP4K2A*	10:22564019	A/C*	all	0.61	0.15	1.11 (0.96–1.29)	0.61	0.28	1.10 (0.93–1.30)	0.62	0.28	1.17 (0.88–1.57)	0.59
				Ph^–^	0.61	0.3	1.09 (0.93–1.28)	0.61	0.36	1.08 (0.91–1.29)	0.62	0.61	1.13 (0.71–1.79)	
				Ph^+^	0.62	0.28	1.20 (0.86–1.67)	0.63	0.63	1.20 (0.57–2.52)	0.62	0.33	1.20 (0.83–1.74)	
rs2836365	*ERG*	21:39768274	A/G*	all	0.3	0.1	1.14 (0.97–1.33)	0.3	0.09	1.17 (0.98–1.40)	0.28	0.76	1.05 (0.77–1.44)	0.27
				Ph^–^	0.3	0.09	1.16 (0.98–1.38)	0.31	0.08	1.18 (0.98–1.41)	0.28	0.82	1.06 (0.64–1.75)	
				Ph^+^	0.27	0.98	1.00 (0.70–1.45)	0.23	0.64	1.22 (0.53–2.83)	0.28	0.83	1.04 (0.70–1.57)	
				Ph^–^/ETV6-RUNX1^–^	0.31	0.08	1.19 (0.98–1.45)	0.32	**0.04**	1.23 (1.01–1.52)	0.28	0.91	1.03 (0.62–1.72)	

Totally 451 patients can be divided into multiple groups: 294 children (16 Ph^+^, and 272 Ph^–^, the rest are not known for Ph status); 157 adults (91 Ph^+^, and 66 Ph^–^). P-values and odds ratios were estimated by the additive logistic regression test, P < 0.05 was bold. Chromosomal locations are based on GRCh37/hg19. Chr, chromosome; RAF, risk allele frequency; ference allele (GRCh37/hg19); ALT, alternative allele; OR, odds ratio; CI, confidence interval. *Asterisk indicates risk allele for ALL susceptibility.

We next performed association analyses in two age groups separately. Interestingly, *ARID5B* and *GATA3* SNPs were only significantly associated with ALL susceptibility in childhood and adolescent/adult patients, respectively (Table 3). Additionally, the independent *IKZF1* signal exhibit its association in both age group, with even higher odds ratio in adult than childhood Ph^–^ patients (1.55 vs. 1.36), while the top *IKZF1* SNP (i.e., rs11978267) was only significant in childhood Ph^–^ patients (Table 3). SNPs at *PIP4K2A* locus also tend to be associated with ALL susceptibility in childhood patients, especially with hyperdiploid subtype [e.g., rs4748793, *P* = 0.02, OR = 1.93 (1.09–3.40)].

**FIGURE 1:**
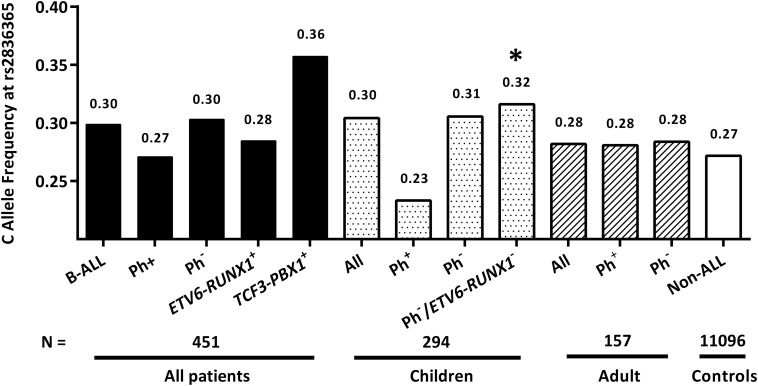
The frequency of *ERG* SNP in age-aged ALL patients. Risk allele frequencies of rs2836365 were illustrated in all patients/non-ALL controls, and separately in each group divided in terms of age and subtypes. Logistic regression tests were conducted by comparing risk allele frequencies of rs2836365 in each patient group with non-ALL controls. ^∗^*P* < 0.05.

Particularly, we focused on the novel Hispanic-specific ALL risk signal at *ERG* locus, observing only marginally significant association of this SNP with ALL susceptibility in childhood patients [*P* = 0.09, OR = 1.17 (0.98–1.40)], but not adults (*P* = 0.76). Because RAF of rs2836365 was observed to be under-represented in patients with *ETV6-RUNX1* fusion (Qian et al., 2018), the association reached significance after excluding *ETV6-RUNX1* and Ph^+^ subtypes of patients [*P* = 0.04, OR = 1.23 (1.01–1.52)], Next, RAF of rs2836365 was determined in each ALL subtype and age group. Consistent with observation in Hispanics (Qian et al., 2018), enriched risk allele was observed in *TCF3-PBX1* subtype at rs2836365 (RAF = 0.36, Figure 1). However, the difference was not significant probably because of the small sample size.

## Discussion

Racial differences in cancer risk and survivals are well documented, but with poor biological basis explored (Schafer and Hunger, 2011). Although increased genomic screenings were conducted in non-Caucasians, the most comprehensive records and novel findings in cancer genomics are still predominated based on Caucasian but limited in Chinese, including ALL.

In this study, we systematically analyzed the association status of the reported top and novel GWAS signals in all-age Chinese ALL patients, demonstrating the large effect of age on genetic predispositions to ALL susceptibility. The top signal at *ARID5B* locus exhibited the strongest association only in childhood patients, which further support its importance in Chinese patients (Wang et al., 2013). However, no significant association was found in adolescents/adults, which is consistent with the findings in Caucasians (Peyrouze et al., 2012; Burmeister et al., 2014), as well as the fact of negative correlation of *ARID5B* SNP effect with age at diagnosis in pediatric patients (Xu et al., 2013). In contrast, only *GATA3* SNP exhibited its significance in adolescents/adults, validating previous Caucasian population based GWAS performed by us and another independent group. On the other hand, we validated the association of *IKZF1*-rs11978267 and *CEBPE* SNP with ALL risk in childhood patients, standing by the side of replication study with positive validation result (Urayama et al., 2018), rather than the race-specific assumption (Wang et al., 2013). Moreover, at least two independent association signals were located at *IKZF1* locus, and higher impact of rs11770117 than rs11978267 was noticed in Hispanics but not Caucasians. In this case, *IKZF1*-rs11770117 was also evaluated in our cohort, and exhibits not only independent association with childhood ALL susceptibility after adjusting for rs11978267, but also marginally significance in adolescents/adults, indicating different regulation patterns of *IKZF1* may be involved in leukemogenesis between children and adults.

Effects of the reported inherited predispositions to ALL varied among different subtypes. For instance, *ARID5B* and *PIP4K2A* SNPs were more enriched in hyperdiploid subtype, which was also validated in our study. However, Ph^+^ subtype has barely mentioned because of its low frequency in childhood ALL. In this study, 70 Ph^+^ patients were enrolled for association analyses, and mostly in adolescents/adults. Although no clear conclusion can be drawn in childhood ALL due to the limited statistical power, we observed significant association of *GATA3* SNP with ALL risk in adults with Ph^+^ subtype, indicating its broad effect in both Ph^+^ and Ph-like ALL in elder patients (Perez-Andreu et al., 2013). To our knowledge, this is the first report that established the correlation of *GATA3* SNP with ALL risk in adult Ph^+^ patients, which therefore requiring more evidences in independent validation cohorts.

Importantly, we recently identified a novel Hispanic-specific signal at *ERG* locus, which is highly related to Native Americans ancestry (Qian et al., 2018). Since ancestors of Native Americans is considered to descend from a single founding population initially split from East Asians (Moreno-Mayar et al., 2018), we sought to evaluate its effect in Chinses ALL patients. Actually, the trend of *ERG*-rs2836365 RAF distribution in each subtype is similar to that in childhood Hispanic patients, which is high in TCF3-PBX1^+^ subtype and low in *ETV6-RUNX1^–^* subtype, while no difference was observed in adults with any subtypes. Association of *ERG*-rs2836365 can only reach statistically significance in childhood patients with Ph^–^/*ETV6-RUNX1^–^*, probably because of the small sample size.

Collectively, our results not only described association of the top GWAS hits with ALL susceptibility in all-age Chinese patient (including Ph^+^ patients), but also provided independent confirmation that the novel signal at *ERG* locus conferred risk of childhood ALL in an age and subtype specific manner. Additionally, the fact of consistent significant race-specific association of *ERG* and *IKZF1*-rs11770117 with ALL susceptibility, suggests the similarity of East Asians and Hispanics for leukemogenesis. However, since the relatively small sample size in our study, additional independent cohorts with larger sample size are needed to confirm the effect of *ERG* SNP, especially in different molecular subtypes.

## Data Availability Statement

The raw data supporting the conclusions of this article will be made available by the authors, without undue reservation, to any qualified researcher.

## Ethics Statement

The studies involving human participants were reviewed and approved by the Ethics Committee of West China Hospital and West China Second Hospital. Written informed consent to participate in this study was provided by the participants’ legal guardian/next of kin.

## Author Contributions

YS, YiZ, and XL designed and supervised this study. FL, DY, JZ, ZD, and YW conducted the experiments and data analyses, and interpreted the data. YY, YQ, WZ, YaZ, BY, LW, and JG collected the clinical information. XL contributed to the conception of the study and drafted the manuscript. All authors contributed to writing of the manuscript and approved the final manuscript.

## Conflict of Interest

The authors declare that the research was conducted in the absence of any commercial or financial relationships that could be construed as a potential conflict of interest.
